# The Death Literacy Index: translation, cultural adaptation, and validation of the Chinese version

**DOI:** 10.3389/fpubh.2023.1140475

**Published:** 2023-05-11

**Authors:** Sok Leng Che, Xiang Li, Mingxia Zhu, Wai I Ng

**Affiliations:** ^1^Nursing and Health Education Research Centre, Kiang Wu Nursing College of Macau, Macao SAR, China; ^2^Education Department, Kiang Wu Nursing College of Macau, Macao SAR, China

**Keywords:** death literacy, Death Literacy Index, validity, reliability, factor analysis

## Abstract

**Objective:**

Applying public health approaches to address palliative care allows for a broader perspective. The Death Literacy Index (DLI) is a novel instrument designed to assess the knowledge and skills required to access, comprehend, and make informed decisions regarding end-of-life care. Translation of the DLI could strengthen the capacity to build desirable services and policies regarding dying and death. It could also help to identify the barriers to services and future advocacy efforts.

**Methods:**

The DLI was forward translated into Chinese and backward translated through two panels. Two rounds of cognitive interviews and a pilot test were conducted before the survey. A sample of 3,221 participants was recruited via an online survey in five cities in southern China (Guangzhou, Zhuhai, Jiangmen, Hong Kong and Macao) to evaluate the factor structure, validity and reliability of the translated DLI. Additionally, multi-group confirmatory factor analyses (MGCFA) were performed to examine measurement invariance across genders and the experiences of parental death.

**Results:**

Exploratory factor analysis showed a six-factor structure for the translated DLI, and confirmatory factor analysis confirmed the structure. The overall scale and subscales had high internal consistency and satisfactory validity. The results from MGCFA showed that death literacy was adequately invariant for different genders and experiences of parental death.

**Conclusion:**

The Chinese DLI is a reliable and valid instrument for measuring death literacy among people in southern China, and therefore can be used for both research and community practice.

## 1. Introduction

In addition to social systems such as health and education, death has a unique system of its own. Kastenbaum initially proposed the concept of the death system in 1977. It consists of five elements: people, places, times, objects, and symbols (1). It is common to consider death and dying as the end result of medical intervention, which involves the dying person, his or her family, medical personnel, the hospital and those responsible for handling the after-death arrangements. Through aggressive and sometimes futile treatments, dying has become medicalized part of the public sphere. In particular, dying is mostly categorized as a matter of concern in the healthcare sector. Palliative care has been endorsed as a means of combating the medicalization of dying and ensuring dignity for the dying (2). Kellehear connected palliative care to public health (3), enabling the possibility of a health promotion approach to dying. In such health promotion discourses, people who are dying have the right and responsibility to control their treatments and information acquisition, which are often delegated to their family (4, 5). The World Health Organization (WHO) also recognized the importance of integrating palliative care into public health systems (6). People who are dying, healthcare professionals with different specialties, and the entire community are connected to death and dying with strong social ties. For instance, health care is provided to the dying person by professionals according to the person's values and preferences, and to help the family cope with challenges associated with the end of life. The dying process may happen at home if it is the preference of the dying person. In that case, a person's social network is also changed.

The aging population is increasing rapidly around the globe (7). This results in an increased number of deaths and rising demand for palliative care services (8, 9). However, Chinese society faces a number of barriers with regard to end-of-life care as well as public awareness of end-of-life care, including inadequate educational and clinical resources and, lack of support from the national health system (10). The quality of death in China ranks low compared with other countries (11). Although the concept of palliative care was introduced in mainland China in the 1990s, its development has been very slow (12). In 2017, National Health and Family Planning Commission issued guidelines on hospice care and piloted the service in some regions for 2 years (13). This was a milestone in promoting the development of hospice care in mainland China at the policy level. However, healthcare professionals have low awareness regarding end-of-life care and inadequate skills and knowledge to provide such care (14). Although 87% of older adults in China died at home over the past 20 years (15), the general public in China is relatively unaware of end-of-life care. In a survey investigating the awareness of palliative care, approximately 90% of respondents had no prior knowledge of the service (10). Almost 90% of older adults received care from family members rather than professional caregiver before death, and 40% of them died in pain (15). This reflects a significant blank of community end-of-life services, and showed a considerable gap in China to achieve the goal of high-quality death. With limited resources and a rapidly growing aging population, the public's need for end-of-life care is expected to increase.

In contrast, Hong Kong has the longest history of palliative care and offers the most comprehensive end-of-life care in China (16). Its quality of death ranks 22nd among 80 countries (11). In spite of this, Hong Kong faces a number of barriers to palliative care development, including barriers related to politics, economics, sociocultural issues, technological advancements, environmental, and legal issues (17, 18). Although over 30% of Hong Kong people preferred to die at home, over 90% death happened in hospitals (19). In terms of service targets, local palliative care services are limited to patients with cancer, end-stage renal disease, chronic obstructive pulmonary disease, advanced heart failure, and neurological disorders. This disease-oriented palliative care policy results in fragmented services and poor collaboration between hospitals and communities, and between the medical and social sectors (17, 20). Consequently, such policies inhibit the capacity of clinical service delivery as well as public awareness of needs and preferences in terms of these services. Clinicians therefore continue to advocate for the government to develop palliative care policy that is locally appropriate (16).

Macao is still in the early stages of developing end-of-life care. While the first palliative care unit was established in Macao in 2000 with 20 beds to provide care for terminal cancer patients and their families, only 35 hospice beds have been added since then (21, 22). However, the demand for palliative care services outweighs the supply, as evidenced by the number of deaths per year increasing 1.7 times, from 1,338 to 2,320, between 2000 and 2021 (23, 24). In terms of policy regarding end-of-life care and death, the Macao SAR government implemented a 10-year action plan for services for older adults in 2016 (25). While end-of-life care was mentioned in the action plan, only brief references were made regarding strengthening education to raise awareness and expand end-of-life services. Moreover, there is no report of the effectiveness, nor is there any baseline data of the action plan that would help to explain what society's current status is. This raises questions about whether the services provided are adequate and meet the needs of the public. The provision of palliative care in Macao is only available in inpatient setting (21). However, a considerable amount of people would like to be cared at home during the end of life (26). While end-of-life care is included in the government's healthcare agenda, primarily through education and inpatient palliative care, there is a significant gap between the needs of the public and existing services.

As with health literacy, Leonard et al. (27) stated that “death literacy is the knowledge and skills that people need to make it possible to gain access to, understand, and make informed choices about end of life and death care options”. Death involves an extensive public health system that is often overlooked as merely a result of medical treatments. The development of services that meet the needs of citizens requires a comprehensive understanding of the current situation. Through the lens of public health, death literacy plays an important role in policy and service development. On the one hand, the level of death literacy of community residents can specifically reflect the level of relevant knowledge as well as the presence of community services that can provide specific guidance for public policy development. On the other hand, death literacy is shaped by personal experiences of interaction with end-of-life care services. Therefore, the promotion of death literacy requires proactive engagement from every sector (28). A measurement tool for death literacy that is applicable to China is urgently needed.

The Death Literacy Index (DLI) was developed to measure the death literacy among the general public in communities or countries, and to provide insight into further intervention development (29). It can inform the healthcare system about education, service provision, and community development to provide support for people who are dying and their families. The DLI is a novel instrument and has only been evaluated in Australia, Turkey, the UK and Sweden (29–32). In the original development (29), the 29-item DLI was identified as having a four-factor structure; two of these factors contained two subscales, resulting in six subscales. All subsequent studies also identified 6 factors that had good internal consistency with the overall scale (Cronbach's alpha = 0.90–0.94) and subscales (Cronbach's alpha = 0.68–0.94) (30–32).

Since the DLI was designed for intervention assessment, it can be used to compare changes before and after the development of the death system, and the public's awareness of death-related information and services (27). It may also assist in identifying barriers to the provision of death services and in developing future advocacy strategies. The Greater Bay Area of China is being promoted by the Chinese government with the aims of facilitating interaction and communication in daily life, and collaboration in economic development in Guangdong Province, Hong Kong, and Macao. Therefore, the objective of this study was to translate the DLI into Chinese and evaluate its applicability in southern Chinese sociocultural contexts, including Macao SAR, Hong Kong SAR, Guangzhou, Zhuhai, and Jiangmen. These cities of southern China share a similar historical and cultural context but have different levels of economic and medical service development. The applicability of the DLI in cities with disparities in medical service accessibility can also be observed.

## 2. Methods

### 2.1. Study design

Using a cross-sectional online survey design, this study recruited residents from five cities in southern China, including Macao, Hong Kong, Guangzhou, Zhuhai, and Jiangmen.

### 2.2. Translation and cultural adaptation of the DLI

The DLI includes four subscales with 29 items in total. The four subscales are practical knowledge, experiential knowledge, factual knowledge, and community knowledge. All items are measured on a five-point Likert scale. A person's practical knowledge refers to the perception of how well he or she can communicate with friends and family about death. This subscale comprises 8 items that include talking support and hands on support (1 point = not at all able, 5 points = very able). The experiential knowledge subscale measures a person's wisdom and skills gained from direct experiences with caring for someone at the end-of-life or death education. Participants were asked to recall their previous loss experience with 5 items (1 point = very untrue of me, 5 points = very true of me). The factual knowledge subscale assesses the understanding of the death system and information required for good planning for the end of one's life using 7 items (1 point = strongly disagree, 5 points = strongly agree). There are 9 items in the community knowledge subscale, which measures a person's knowledge of accessing support for people who are dying and their caregivers (1 point = strongly disagree, 5 points = strongly agree). There are no reverse-coded items. Scores are calculated by summing items and scaling the number of items in a subscale (with a range of scores between 0 and 10). Authorization to its use was obtained from the authors.

The translation process of the DLI followed WHO translation guidelines (33), including forward translation, expert panel back-translation, pretesting and cognitive interviewing. Forward translation was conducted by two independent, bilingual, fluent Cantonese and English local translators. The translators were instructed to use plain and conceptually equivalent language to translate. After forward translation, a panel was convened to discuss the differences between the two translations and between the translators and the research team, and a consensus was reached. Panel members included the two translators, the principal investigator (WIN), and three other research members (SLC, XL, and MZ). During the panel, there were discussions about cultural applicability regarding specific words, such as “emotional strength” in Item 9, “compassionate” in Item 12, and “cemetery staff” in Item 20. All disagreements were resolved during the panel. The members decided to change “cemetery staff” to “cemetery/funeral staff”. Most deceased persons are not currently buried in a cemetery, but are kept in a shrine after cremation. Additionally, “culturally appropriate support” in Item 24 was modified to “support in line with Chinese culture”. In addition, Hong Kong and Macao use traditional Chinese, while mainland China uses simplified Chinese. Therefore, two different versions of the questionnaire were used. Furthermore, the word “quality” used in Mainland China and in Hong Kong and Macao is different, so we applied the equivalent words in traditional and simplified Chinese versions. After the translated version was finalized, two other translators were invited to conduct back-translation using the same approach as forward translation.

Cognitive interviews were used to identify phrases or terms that could lead to ambiguity (34). Two rounds of cognitive interviews were conducted in October 2022 after the translated DLI was finalized, with 10 participants in each round. The cognitive interviewees were recruited through acquaintances of the research team, to ensure the diversity of the participants. The inclusion criteria for the cognitive interviews were Chinese individuals aged 18–74 years who lived in Macao, Hong Kong, Guangzhou, Zhuhai, or Jiangmen. The recruited participants had diverse backgrounds, included both males and females, and of different ages, education levels and occupations. In practice, the research team members held a consensus meeting regarding the interview protocol before the interviews, to agree on the interview outline and to align the cues for follow-up questions. Individual interviews were performed by the research team for each participant. The interviews began with the collection of basic demographic information. Concurrent verbal probing was administered following the interview guide which created by the research team (35). Participants were asked to verbally describe their interpretation after they answered each item. When the interviewee's description differs from the original intention of the item, it will be recorded on paper along with the suggestions for modification. Modification of the items was made before the second round of cognitive interviews that took into account the feedback from the first round. Interview protocol was not adjusted after the first round.

The cognitive interviews resulted in the modification of words on Items 11, 15, 19, and 20. In Item 11, the word “developed” was changed to “increased” because participants stated that wisdom cannot be developed. In Item 15, participants suggested that “planning” should be changed to “preparing” because “planning for death” might remind people of “planning for suicide” in the context of the Chinese language. In Item 19, participants found it difficult to connect “illness trajectory” at the beginning and “quality of end of life” at the end since not all illnesses are life-threatening. A conditional sentence was therefore added to indicate the situation of being seriously ill. In Item 20, the participants' opinion was that the word “contribution” implied too much credit, so it was changed to “help” (Supplementary material 1 provides the original and Chinese version).

After the cognitive interviews, the research team conducted a pilot test to examine the feasibility of the translated DLI and to identify possible alternative expressions in the Chinese population. A minimum sample size of 30 from the population of interest is generally recommended for a pilot study (36). Participants were purposively recruited in the five cities through acquaintances of the research team. Fifty-three participants aged 22–74 years (41.0 ± 15.0) were recruited in the pilot test. Cronbach's alpha of the translated DLI was 0.88 for the total scale. The final translated version of the DLI contained the same number of items as the original version.

### 2.3. Questionnaire and participant recruitment

Survey data were collected using a structured online questionnaire. The questionnaire contained the translated DLI and sociodemographic characteristics of participants, including their age, gender, level of education, marital status, religious beliefs, occupation, whether they had children and siblings, and whether their parents were alive.

Inclusion criteria were people who were residing in the abovementioned five cities at the time of the survey implementation and who identified themselves as Chinese, were aged 18–74, were able to give consent and understood written Chinese. Participant recruitment and data collection were conducted from October to November 2022. A sample size of ten respondents was calculated for each item in the DLI (37). Therefore, the target number of recruitments was at least 290 participants. Convenience and snowball sampling methods were applied via online advertisements and social media platforms. Posters with traditional and simplified Chinese, a short description of the study, and the link to the questionnaire were distributed to various local social service organizations and colleges via social media platforms such as Facebook, WhatsApp and WeChat. Potential participants could click on the link to provide informed consent and proceed with the questionnaire. After completing the questionnaire, participants were encouraged to distribute the study information to their friends and others who were interested.

### 2.4. Statistical analysis and scale evaluation

Raw data were coded using Microsoft Office Excel 2013, confirmatory factor analysis (CFA) was performed using Amos (version 22.0), and Statistical Package for the Social Sciences Version 22 (SPSS, version 22) was utilized for data manipulation and other analyses. Analyses were restricted to respondents who completed the full questionnaire (*n* = 3,221). The threshold for statistical significance was set to *p* < 0.05.

All demographic characteristics were categorized and were calculated as frequencies and percentages. Item analysis was conducted to examine the quality of the DLI items. A distribution analysis was conducted to determine interpretability (median, range, interquartile range). To discriminate participants with the highest or lowest possible score for the item, floor and ceiling effects were evaluated, which indicated whether 15% of respondents scored the lowest or highest possible (38, 39).

Exploratory factor analysis (EFA) was implemented to test dimensionality and internal consistency, and CFA was used to confirm whether the factor structure of the translated DLI matched the EFA results. The dimensionality of EFA was set to extract factors with eigenvalues > 1 using principal component analysis (PCA) with varimax rotation. Rotated factor loadings loaded on the primary factor > 0.4 were considered satisfactory (40). Parallel analysis (PA) was performed to compare the eigenvalues. To assess the suitability of the data for factor analysis, Kaiser-Meyer-Olkin (KMO) measurement and Bartlett's test of sphericity were used. The dataset was considered appropriate for PCA when KMO was over 0.70 and Bartlett's test of sphericity was significant (*p* < 0.05) (37). The internal consistency was examined by Cronbach's alpha and McDonald's omega, and composite reliability (CR) and average of variance extracted (AVE) were examined to confirm discriminant and convergent validity. Model with CR > 0.7 and AVE > 0.5 were considered adequate (41).

Regarding CFA, the goodness of fit and acceptability of the model were assessed by indicators such as the comparative fit index (CFI), goodness-of-fit index (GFI), non-norm-fitting index (Tucker-Lewis Index, TLI), root mean square error of approximation (RMSEA), and standardized root mean square residual (SRMR). The model was considered to have reasonable fit and acceptability if the CFI > 0.9, GFI > 0.9, TLI > 0.9, RMSEA <0.08, and SRMR <0.09, using the maximum-likelihood method (42). Studies have reported that there are differences in death anxiety and coping strategies between genders and whether the individuals have had loss experiences (43, 44). Also, the research team assumed that with the experience of parental death, the individual would have more interaction with community services and health system regarding end-of-life care. Therefore, after the measurement model was confirmed, multi-group confirmatory factor analysis (MGCFA) was performed to investigate validity across different genders and experiences with parental death. Three levels of measurement invariance were tested, i.e., configural measurement invariance, metric invariance, and scalar measurement. If the changes in CFI (ΔCFI), TLI (ΔTLI), RMSEA (ΔRMSEA) and SRMR (ΔSRMR) were <0.01, the model was considered acceptable (45).

### 2.5. Ethical approval

Ethical approval for the study was granted by the Research Management and Development Department of Kiang Wu Nursing College of Macau (reference: 2021DEC02). All participants were informed about the purpose of the study and their right to withdraw from the study at any time. Informed consent was obtained from all participants who agreed to participate.

## 3. Results

### 3.1. Participant characteristics

There were 3,221 valid responses in the questionnaire survey study. The majority were female (79.2%) and, aged 18 to 34 (72.6%) with a mean age of 28.3 ± 12.4 years (range 18–74). Most of them were students (52.8%), had an education level of college or above (90.9), were not married (65.9%), and had siblings (81.8%) but did not have children (71.8%) or religious belief (73.1%) (Table 1).

**Table 1:** Socio-demographic characteristics (*n* = 3,221).

**Variable**	***n***	**%**
**Gender**		
Male	671	20.8
Female	2,550	79.2
**Age (year)**		
18–34	2,338	72.6
35–54	734	22.8
55–74	149	4.6
**Education level**		
Primary school or below	36	1.1
Secondary school	257	8.0
College or above	2,928	90.9
**Marital status**		
Not married	2,124	65.9
Married/cohabited	996	30.9
Separated/divorced	82	2.5
Widowed	19	0.6
**Children**		
Yes	909	28.2
No	2,312	71.8
**Siblings**		
Yes	2,636	81.8
No	585	18.2
**Religious beliefs**		
Yes	867	26.9
No	2,354	73.1
**Occupation**		
Medical (assistant) professional	767	23.8
Student	1,702	52.8
Other	587	18.2
Not employed	165	5.1
**Experience of parental death**		
Both parents alive	2,616	81.2
At least one parent died/ don't know	605	18.8

### 3.2. Item and distribution analysis

The item discrimination test showed positive discrimination for all of the items (46) (Table 2). The result of distribution analysis showed that the mean score of each subscale and the DLI total mean score represented the possible ranges. Applying the criterion of 15% of participants scoring the lowest or highest possible score, the DLI total mean score did not show any floor or ceiling effects. In terms of subscales, a ceiling effect was demonstrated in the “doing hands on care” and “experiential knowledge” subscales, with over 15% of participants obtaining the highest possible score (Table 3).

**Table 2:** Item analysis of the translated DLI (*n* = 3,221).

**Item**	**Mean**	**SD**	**Skewness**	**Item discrimination**	**Cronbach's α if item deleted**	**Corrected item-total correlation coefficients**
1. Talk about death, dying or grieving to a close friend	7.5	2.35	−1.13	−17.66^***^	0.94	0.34
2. Talk about death, dying or grieving to a child	6.9	2.63	−0.86	−21.07^***^	0.94	0.36
3. Talk to a newly bereaved person about their loss	5.0	3.15	−0.04	−24.43^***^	0.94	0.34
4. Talk to a GP about support at home or in their place of care for a dying person	7.5	2.26	−1.13	−18.25^***^	0.94	0.35
5. Feeding a person or assisting them to eat	8.3	1.81	−1.22	−16.92^***^	0.94	0.38
6. Bathing a person	7.6	2.26	−1.03	−22.31^***^	0.94	0.41
7. Lifting a person or assisting to transfer them	8.0	2.02	−1.21	−19.57^***^	0.94	0.41
8. Administering injections	7.2	2.74	−0.97	−22.55^***^	0.94	0.39
9. Increased my emotional strength to help others with death and dying processes	7.2	2.20	−0.85	−30.51^***^	0.94	0.57
10. Led me to re-evaluate what is important and not important in life	7.6	2.01	−0.97	−23.26^***^	0.94	0.48
11. Increased my wisdom and understanding	7.5	2.05	−0.91	−28.43^***^	0.94	0.55
12. Made me more compassionate toward myself	7.6	2.03	−0.97	−26.78^***^	0.94	0.54
13. Provided me with skills and strategies when facing similar challenges in the future	7.4	2.08	−0.88	−30.19^***^	0.94	0.58
14. I know the law regarding dying at home	5.6	2.74	−0.30	−45.40^***^	0.94	0.64
15. I feel confident in knowing what documents you need to complete in preparing for death	5.8	2.73	−0.40	−46.63^***^	0.94	0.66
16. I know how to navigate the health care system to support a dying person to receive care	6.5	2.56	−0.75	−46.40^***^	0.94	0.70
17. I know how to navigate funeral services and options	6.2	2.66	−0.57	−46.98^***^	0.94	0.68
18. I know how to access palliative care in my area	6.0	2.70	−0.48	−49.64^***^	0.94	0.69
19. When I am seriously ill, I have sufficient understanding of illness trajectories to make informed decisions around medical treatments available and how that will shape quality of end of life	7.1	2.28	−0.98	−31.40^***^	0.94	0.59
20. I know what the cemetery staff/ funeral staff can help at end of life	6.7	2.40	−0.86	−38.57^***^	0.94	0.66
21. Access community support	6.2	2.60	−0.56	−58.02^***^	0.94	0.75
22. Provide day to day care for the dying person	6.3	2.55	−0.66	−53.96^***^	0.94	0.74
23. Access equipment required for care	6.3	2.57	−0.62	−54.70^***^	0.94	0.75
24. Access appropriate support in line with Chinese culture	6.2	2.60	−0.57	−52.33^***^	0.94	0.72
25. Access emotional support for myself	6.5	2.46	−0.77	−45.98^***^	0.94	0.72
26. People with life threatening illnesses	6.2	2.62	−0.64	−44.56^***^	0.94	0.67
27. People who are dying	6.2	2.64	−0.62	−44.67^***^	0.94	0.66
28. Carers for people who are dying	6.2	2.65	−0.62	−44.17^***^	0.94	0.66
29. People who are grieving	6.2	2.64	−0.61	−42.76^***^	0.94	0.65
Total DLI	6.7				0.94	

*** *p* < 0.001.

All items ranged 0–10.

**Table 3:** Distribution analysis of the translated DLI (*n* = 3,221).

**Subscales**	**Mean (SD)**	**Median**	**IQR**	**Floor and ceiling effect**	
				**Lowest possible total score *n* (%)**	**Highest possible total score *n* (%)**
**Practical knowledge**	**7.25 (1.58)**	**7.50**	**1.88**	**10 (0.3%)**	**175 (5.4%)**
Talking support	6.73 (1.99)	7.50	1.88	35 (1.1%)	272 (8.4%)
Doing hands on care	7.78 (1.86)	7.50	2.50	20 (0.6%)	792 (24.6%)
**Experiential knowledge**	**7.45 (1.79)**	**7.50**	**2.00**	**17 (0.5%)**	**526 (16.3%)**
**Factual knowledge**	**6.27 (2.07)**	**6.79**	**2.50**	**24 (0.7%)**	**205 (6.4%)**
**Community knowledge**	**6.26 (2.26)**	**7.50**	**2.50**	**43 (1.3%)**	**242 (7.5%)**
Accessing help	6.29 (2.36)	7.50	2.50	67 (2.1%)	293 (9.1%)
Support groups	6.21 (2.54)	7.50	2.50	109 (3.4%)	341 (10.6%)
**DLI total**	**6.74 (1.50)**	**6.90**	**1.81**	**5 (0.2%)**	**80 (2.5%)**

SD, Standard deviation; IQR, interquartile range.

All items ranged 0–10.

### 3.3. Exploratory factor analysis

The KMO was 0.948, and Bartlett's test of sphericity was significant ($\chi_{406}^{2}$ = 80,632.31; *p* < 0.001), suggesting that the matrix was suitable for factor extraction. PA suggested five factors, instead of six factors in the original development and later adaptation studies. However, all of the items in the “support groups” subscale (Items 21–25) showed cross-loading on “factual knowledge” and on “community knowledge”. It has been reported that the result of PA may not be satisfactory when factors are highly correlated (47). Correlation analysis was then performed on all six subscales and showed that “accessing help” had strong correlation with “support groups” and “factual knowledge” (Table 4). Additionally, the CFA of the five-factor structure suggested that it was a poor-fitting model [$\chi_{367}^{2}$ = 15,015.058 (*p* < 0.001), CFI = 0.818, GFI = 0.656, TLI = 0.801, RMSEA = 0.111, SRMR = 0.111]. Therefore, the research team continued the subsequent analysis using the six-factor structure.

**Table 4:** Correlation matrix of six-factor translated DLI (*n* = 3,221).

	**F1**	**F2**	**F3**	**F4**	**F5**	**F6**
F1: Talking support	1					
F2: Doing hands on care	0.35^**^	1				
F3: Experiential Knowledge	0.47^**^	0.47^**^	1			
F4: Factual Knowledge	0.32^**^	0.29^**^	0.44^**^	1		
F5: Accessing help	0.19^**^	0.22^**^	0.35^**^	0.73^**^	1	
F6: Support groups	0.10^**^	0.17^**^	0.25^**^	0.59^**^	0.72^**^	1

* *p* < 0.01.

In the six-factor structure, Item 21 “access community support” loaded at 0.41 on the “factual knowledge” and “accessing help” factors. The research team decided to keep it on “accessing help” since it had stronger loading on “accessing help”. The items accounted for a cumulative variance of 74.93%. The overall Cronbach's alpha coefficient of the translated DLI was 0.94, while the subscales were 0.76–0.97, with similar results of Omega coefficients, suggesting good internal consistency (Table 5).

**Table 5:** Exploratory factor analysis and convergent validity of the six-factor translated DLI (*n* = 3,221)^*^.

	**Factor Loading**						**Communalities**
	**F1**	**F2**	**F3**	**F4**	**F5**	**F6**	
15. I feel confident in knowing what documents you need to complete in preparing for death	0.80						0.75
17. I know how to navigate funeral services and options	0.79						0.75
14. I know the law regarding dying at home	0.74						0.69
16. I know how to navigate the health care system to support a dying person to receive care	0.72						0.70
18. I know how to access palliative care in my area	0.69						0.68
20. I know what the cemetery staff/ funeral staff can help at end of life	0.54						0.55
19. When I am seriously ill, I have sufficient understanding of illness trajectories to make informed decisions around medical treatments available and how that will shape quality of end of life	0.53						0.49
27. People who are dying		0.88					0.94
28. Carers for people who are dying		0.88					0.94
26. People with life threatening illnesses		0.86					0.91
29. People who are grieving		0.85					0.90
22. Provide day to day care for the dying person			0.77				0.87
23. Access equipment required for care			0.77				0.88
24. Access appropriate support in line with Chinese culture			0.76				0.85
25. Access emotional support for myself			0.75				0.80
21. Access community support	0.41		0.71				0.83
11. Increased my life wisdom and understanding				0.85			0.81
12. Made me more compassionate toward myself				0.84			0.79
13. Provided me with skills and strategies when facing similar challenges in the future				0.82			0.79
10. Led me to re-evaluate what is important and not important in life				0.77			0.70
9. Increased my emotional strength to help others with death and dying processes				0.72			0.67
7. Lifting a person or assisting to transfer them					0.85		0.80
5. Feeding a person or assisting them to eat					0.83		0.80
6. Bathing a person					0.81		0.71
8. Administering injections					0.72		0.59
2. Talk about death, dying or grieving to a child						0.82	0.73
1. Talk about death, dying or grieving to a close friend						0.79	0.71
4. Talk to a GP about support at home or in their place of care for a dying person						0.70	0.63
3. Talk to a newly bereaved person about their loss						0.60	0.49
Eigenvalues	4.61	3.97	3.84	3.83	3.03	2.45	
% of Variance	15.91	13.69	13.25	13.21	10.44	8.44	
% of Cumulative Variance	15.91	29.60	42.85	56.05	66.49	74.93	
Cronbach's alpha (α)/ McDonald's omega (ω)	0.91/0.91	0.97/0.97	0.96/0.96	0.91/0.91	0.85/0.85	0.76/0.76	0.94/0.94
Construct Reliability (CR)	0.91	0.97	0.96	0.92	0.87	0.79	0.98
Average of variance extracted (AVE)	0.58	0.90	0.81	0.69	0.63	0.49	0.68

^*^Only loadings ≥ 0.40 in the items are shown in the table.

F1, Factual Knowledge; F2, Community support group; F3, Accessing help; F4, Experiential Knowledge; F5, Hands on support; F6, Talking support.

### 3.4. Confirmatory factor analysis

**Figure 1:**
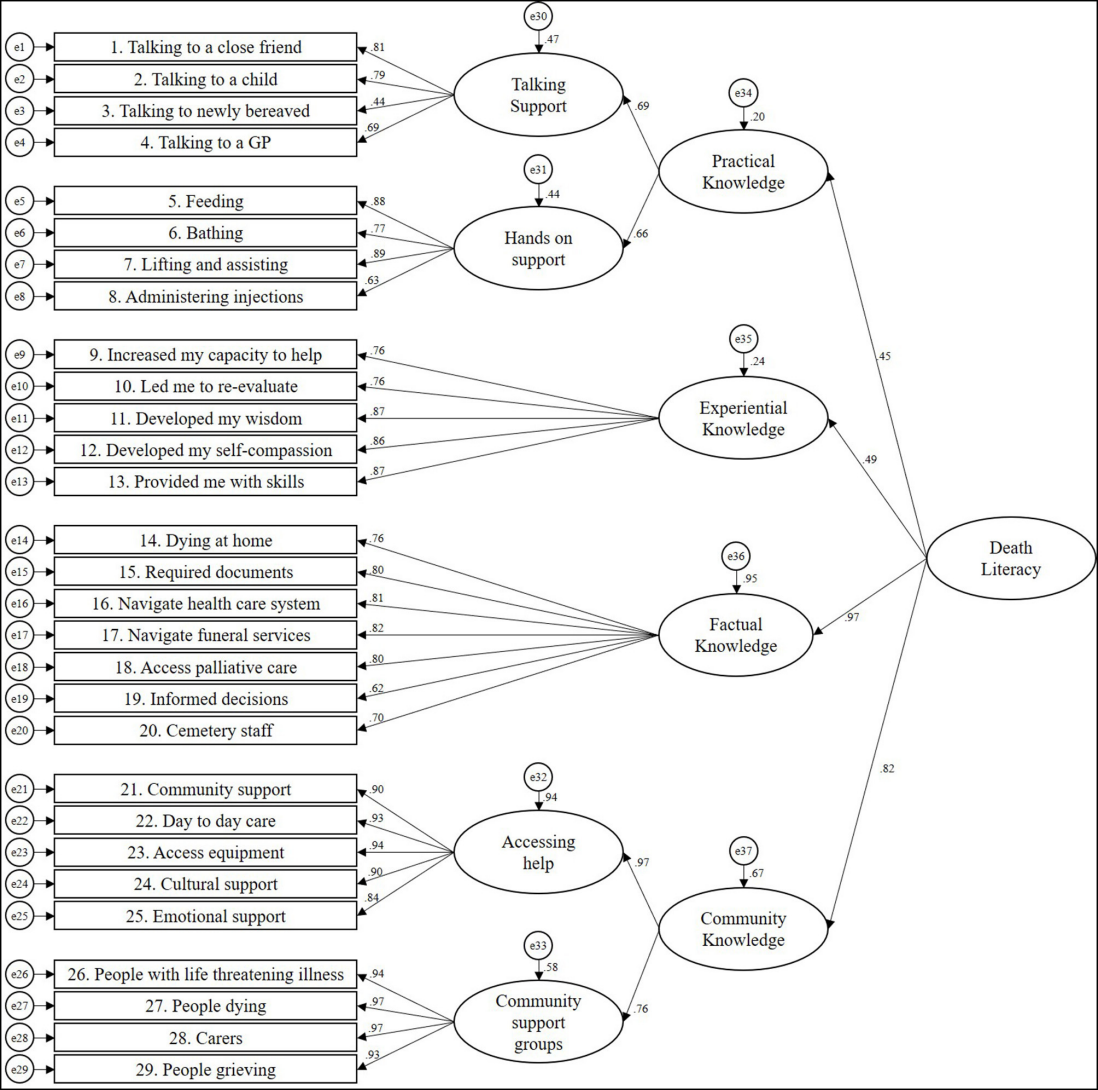
Structural equation model for the fitting model for the Chinese translated Death Literacy Index.

In the CFA results, the model fit for the six-factor structure of the translated DLI was as follows: $\chi_{367}^{2}$ = 3,889.860 (*p* < 0.001), CFI = 0.956, GFI = 0.915, TLI = 0.952, RMSEA = 0.055 (90% C.I. = 0.053–0.056) and SRMR = 0.0513 (Table 6). All scales had CR > 0.7 and AVE > 0.4, indicating adequate discriminant and convergent validity. The factor loading of the items in CFA ranged from 0.44 to 0.97. The path diagram for the CFA model is shown in Figure 1. In MGCFA, the results showed good fit in the values of CFI, TLI, RMSEA and SRMR for both comparisons of gender and experience of parental death (Table 7).

**Table 6:** Confirmatory factor analysis between full sample and sub-groups.

	**CFI**	**GFI**	**TLI**	**RMSEA (90% CI)**	**SRMR**
Full sample (*n* = 3,221)	0.956	0.915	0.952	0.055 (0.053, 0.056)	0.0531
Male (*n* = 671)	0.937	0.864	0.931	0.066 (0.063, 0.070)	0.0650
Female (*n* = 2,550)	0.954	0.910	0.949	0.056 (0.054, 0.058)	0.0522
Both parents alive (*n* = 2,616)	0.957	0.912	0.953	0.055 (0.053, 0.057)	0.0554
At least one parent died/ don't know (*n*=605)	0.931	0.872	0.924	0.064 (0.060, 0.068)	0.0583

**Table 7:** Multi-group confirmatory factor analysis for different sub-groups.

	**CFI**	**TLI**	**RMSEA (90% CI)**	**SRMR**	**Model compare**	**ΔCFI**	**ΔTLI**	**ΔRMSEA**	**ΔSRMR**	**Decision**
**Gender**										
M1: Configural invariance	0.950	0.945	0.041 (0.040, 0.042)	0.0650						
M2: Metric invariance	0.950	0.946	0.041 (0.040, 0.042)	0.0648	M1	0	0.001	0	−0.0002	Accept (Δ <0.01)
M3: Scalar invariance	0.949	0.947	0.041 (0.039, 0.042)	0.0648	M2	−0.001	0.001	0	0	Accept (Δ <0.01)
**Experience of parental death**										
M4: Configural invariance	0.953	0.948	0.040 (0.039, 0.041)	0.0554						
M5: Metric invariance	0.953	0.949	0.040 (0.039, 0.041)	0.0548	M4	0	0.001	0	−0.0006	Accept (Δ <0.01)
M6: Scalar invariance	0.951	0.950	0.040 (0.038, 0.041)	0.0547	M5	−0.002	0.001	0	−0.0001	Accept (Δ <0.01)

## 4. Discussion

The Chinese version of the DLI demonstrated a satisfactory measure of validity and reliability for assessing the Chinese general public living in southern China. To our knowledge, this is the first study to validate the DLI in Asia and to translate the DLI into Chinese. Another validation effort is underway in northern China (Beijing).

The lowest loading items were informed decisions (Item 19), cemetery staff (Item 20), and talking to newly bereaved (Item 3). The low homogeneity of the results on the informed decisions item suggested that people may be less aware of the importance of the illness trajectory, which may be the focus of future education. In the translation process, “funeral workers” was added to the cemetery staff item, and “help” rather than “contribution” was suggested in cognitive interviews. Funeral practitioners have been stigmatized and marginalized in Chinese culture (48), and after-death rituals are diversified between ethnic groups. Therefore, people might have different impressions of the contribution and benefits of bereavement outcomes that funeral practitioners can bring to the family (49). Furthermore, the barriers to discussing life-threatening illnesses and bereavement may also be influenced by traditional Chinese sociocultural factors, such as “family determination” and “death as taboo” (50). Both family members and healthcare professionals tend not to involve patients in the clinical decision-making process (51, 52). Despite this, the internal consistencies of factual knowledge and talking support were good.

In the six-factor structure, item 21 showed cross-loading in EFA on the “factual knowledge” and “accessing help” subscales. This might be caused by the fact that when people need information to access community resources, the information is a kind of fact. However, because the loading only just meets the threshold of 0.4, this is unlikely to be a significant concern. Regarding internal reliability, although the AVE in the “talking support” subscale was <0.5, it is still considered to have adequate convergent validity if the CR is higher than 0.6, as suggested by Fornell and Larcker (41).

The mean scores of the DLI scales and subscales in the current study were consistently higher than those in the Australian and UK populations (27, 30). This suggests that the levels of death literacy reported in the Chinese population were higher. One prominent difference is that the sample in this study was significantly younger than that in the UK study. Studies have found that older age and more knowledge of end-of-life care are associated with more positive death attitudes (53). The younger generation is believed to have less experience and knowledge of death. However, the younger generation was reported more death anxiety compare to the middle-aged (54), this might have led to more information seeking. There is a need to clarify the relationship between age and death literacy, as well as other influencing factors. Furthermore, considering this study was conducted during the COVID-19 pandemic in China, most people were probably confronted with their own mortality more than before the pandemic. With new death cases reported every day, people might have become alarmed by the impermanence and fragility of life. As a coping strategy for facing the fear of death, people would increase their motivation of health information-seeking behaviors (55, 56). It can be assumed that increased exposure to death might also increase motivation to learn about death, contributing to the high score on the DLI.

Interpretability was found to be good, but a ceiling effect was observed in two subscales, i.e., “doing hands on care” and “experiential knowledge”. The two subscales were also found to have the highest mean scores, suggesting that participants had the most confidence in performing such care, which can be attributed to instrumental support being viewed as filial behaviors when parents are at the end of their lives (57). This kind of reciprocal relationship is appreciated in Chinese communities (58, 59). The “support groups” subscale scored the lowest on the DLI, suggesting that people consider support groups to be inaccessible, and peer support services to be inadequate. The lack of awareness of support groups in the community might also contribute to the low score on the support group subscale. There are sociocultural barriers to the acceptance of peer support groups, and Chinese are reported to be more conservative in regard to self-disclosure (60, 61). Despite this, a variety of community-based peer support models are being developed in China (62, 63), and locally developed peer support groups are becoming more acceptable to Chinese people (64). Because it is difficult to determine the cause of the low score on the “support group” subscale simply by the items of the scale, it may be necessary to include items that reflect community resources in subsequent research. On the other hand, it is worth noting that participants considered death to be positive and purposeful for one's life; this contradicts with traditional Chinese philosophy, which sees death as bad fortune (65). Previous studies have shown that loss experiences could decrease death anxiety, and gender differences can also affect death anxiety and coping strategies (43, 44). By applying MGCFA, this study assessed the measurement invariance of the DLI across genders and the experiences of parental death and showed sufficient invariance. This information allows us to interpret and compare the mean scores of the DLI across genders and participants' experiences of parental death.

The DLI was established to be a valid and reliable indicator of death literacy in southern China, and it may provide valuable information for the development of end-of-life care services for dying people and their families. As death literacy of the general public can reflect their knowledge about the availability and accessibility of death-related support or services in the community, the level of death literacy of the public can be utilized as an evaluation to assess the effectiveness of the interventions of hospice services. To investigate the stability, the authors encourage future studies to validate the Chinese DLI in different target groups, such as different age and professional groups, and in different regions across China. Further studies of death literacy across the lifespan are also warranted, since studies suggest death-related perceptions are changeable at different ages, and with the accumulation of life experience (66, 67). As the DLI is beginning to be used in different countries, it can facilitate collaborations across professionals to develop a death system for supporting individuals and their families during end-of-life care and grieving periods.

The current study has a few strengths. First, this study involved five cities in southern China, each representing a different level of economic development and cultural background. Second, the items of the Chinese DLI were culturally adapted and are suitable for populations with southern Chinese culture. Although the sample size in this study is large, the disproportionate proportion of young adults in the sample should be taken into account in the process of generalization and interpretation. Moreover, the sample composition had a large proportion of females and college students. The exploitation of the convenience sampling method may hinder sample representation. It is recommended that future validation studies include a greater number of older people, males, and working individuals, to improve the representativeness of these groups. Additionally, the Chinese DLI has not been compared with other scales, such as the Death Anxiety Scale (68) and the Palliative Care Knowledge Scale (69). Therefore, it is recommended that further analysis be conducted to differentiate the Chinese DLI from other measurement tools, to identify different concepts relating to death in the Chinese population. The content validity of the translated DLI was not reviewed by an expert panel in the field; it was reviewed only by the translation panel. This gap will be addressed in future studies. Finally, this study only examined the effect of parental death on the DLI. However, other experiences of loss might also affect death literacy. It is suggested that future studies to explore the relationship between experiences of loss of other significant others and death literacy.

## 5. Conclusion

It is expected that the increased needs for end-of-life care among people in Chinese society will prompt the government to devote more resources into the health and social care system. The current study demonstrated that the Chinese DLI is a valid and reliable tool for death literacy assessment among the southern Chinese population. Therefore, the Chinese DLI can be used to identify public needs for end-of-life care services, and to measure the effectiveness intervention development for people of southern China.

## Data availability statement

The raw data supporting the conclusions of this article will be made available by the authors, without undue reservation.

## Ethics statement

The studies involving human participants were reviewed and approved by the Research Management and Development Department of Kiang Wu Nursing College of Macau. The participants provided their written informed consent to participate in this study.

## Author contributions

Conceptualization and investigation: SLC, WIN, XL, and MZ. Methodology: SLC, WIN, and XL. Formal analysis, data curation, writing—original draft preparation, and visualization: SLC. Writing—review and editing: WIN, XL, and MZ. Supervision, project administration, and funding acquisition: WIN. All authors have read and approved the submitted version.

## Supplementary material

The Supplementary Material for this artilce can be found online at: https://www.frontiersin.org/articles/10.3389/fpubh.2023.1140475/full#supplementary-material
