# Supplementary material for: The Death Literacy Index: translation, cultural adaptation, and validation of the Chinese version
Source: Front Public Health. 2023 May 11;11:1140475. doi: 10.3389/fpubh.2023.1140475 (PMC10213892; doi:10.3389/fpubh.2023.1140475)
Supplement: Supplementary file 1 [file Data_Sheet_1.DOCX]

**Supplement 1**

**Comparison of the original and Chinese version Death Literacy Index***

| Original version | Chinese version (in English) | Chinese version (in Chinese) |
| --- | --- | --- |
| **Practical Knowledge** (2 Subscales) | **Practical Knowledge (2 subscales)** | **實踐知識（2個分量表）** |
| **Talking support** | **Talking support** | **談論支持（1）** |
| Please rate how difficult or easy you would find the following talking support (on a scale of 1-5 between *Not at all able* to *Very able*) | Please rate how difficult or easy you would find the following talking support (on a scale of 1-5 between *Not at all able* to *Very able*) | 以下是關於談論支持的題目，請按您覺得自己能夠做到的支持程度評分（在1-5的範圍內，從*完全不能夠*到*完全能夠*） |
| 1. Talk about death, dying or grieving to a close friend | 1. Talk about death, dying or grieving to a close friend | 1. 與親密的朋友談論死亡、臨終或哀傷 |
| 2. Talk about death, dying or grieving to a child | 2. Talk about death, dying or grieving to a child | 2. 與孩子談論死亡、臨終或哀傷 |
| 3. Talk to a newly bereaved person about their loss | 3. Talk to a newly bereaved person about their loss | 3. 與剛失去至親的人談論喪親之痛 |
| 4. Talk to a GP about support at home or in their place of care for a dying person | 4. Talk to a GP about support at home or in their place of care for a dying person | 4. 與醫生談論為臨終者在家中或照顧場所提供支持 |
| **Doing hands on care** | **Doing hands on care** | **照護實踐（2）** |
| Please rate how difficult or easy you would find the following hands on support. *Undertake the following care duties for the dying* (on a scale of 1-5 between *Not at all able* to *Very able*) | Please rate how difficult or easy you would find the following hands on support. *Undertake the following care duties for the dying* (on a scale of 1-5 between *Not at all able* to *Very able*) | 以下是關於照護行為的題目，請按您覺得自己能夠為臨終者執行照護實踐的程度評分（在1-5的範圍內，從*完全不能夠*到*完全能夠*） |
| 5. Feeding a person or assisting them to eat | 5. Feeding a person or assisting them to eat | 5. 餵食或協助進食 |
| 6. Bathing a person | 6. Bathing a person | 6. 洗澡 |
| 7. Lifting a person or assisting to transfer them | 7. Lifting a person or assisting to transfer them | 7. 扶抱或協助轉移體位 |
| 8. Administering injections | 8. Administering injections | 8. 執行注射 |
| **Experiential Knowledge** | **Experiential Knowledge** | **經驗知識** |
| Please rate how much each of the below statements sound like you. *My previous experience of grief, loss or other significant life events has* (on a scale of 1-5 between *Very untrue of me* to *Very true of me*) | Please rate how much each of the below statements sound like you. *My previous experience of grief, loss or other significant life events has* (on a scale of 1-5 between *Very untrue of me* to *Very true of me*) | 以下的題目是有關過往的哀傷、喪親或其他生活重大事件的經歷對您的影響，請評價以下陳述與您自身情況的符合程度（在1-5的範圍內，從*非常不符合*到*非常符合*） |
| 9. Increased my emotional strength to help others with death and dying processes | 9. Increased my emotional strength to help others with death and dying processes | 9. 提升了我的情緒調適能力，以幫助其他人面對死亡和臨終的過程 |
| 10. Led me to re-evaluate what is important and not important in life | 10. Led me to re-evaluate what is important and not important in life | 10. 讓我重新評估生命中甚麼是重要和不重要的 |
| 11. Developed my wisdom and understanding | 11. Increased my life wisdom and understanding | 11. 增長了我的見識和人生智慧 |
| 12. Made me more compassionate toward myself | 12. Made me more compassionate toward myself | 12. 對自己更關愛及寬容 |
| 13. Provided me with skills and strategies when facing similar challenges in the future | 13. Provided me with skills and strategies when facing similar challenges in the future | 13. 為我在未來面對相似的挑戰時提供技巧和策略 |
| **Factual Knowledge** | **Factual Knowledge** | **事實知識** |
| Please rate how much each of the below statements sound like you (on a scale of 1-5 between *Strongly disagree* to *Strongly agree*) | Please rate how much each of the below statements sound like you (on a scale of 1-5 between *Strongly disagree* to *Strongly agree*) | 請評價以下陳述與您自身情況的符合程度（在1-5的範圍內，介乎於*非常不同意*至*非常同意*之間） |
| 14. I know the law regarding dying at home | 14. I know the law regarding dying at home | 14. 我知道有關在家中死亡的法律條例 |
| 15. I feel confident in knowing what documents you need to complete in planning for death | 15. I feel confident in knowing what documents you need to complete in preparing for death | 15. 我有信心知道為死亡做準備時所需要完成的文件 |
| 16. I know how to navigate the health care system to support a dying person to receive care | 16. I know how to navigate the health care system to support a dying person to receive care | 16. 我知道如何尋求醫療照護系統的支援，以支持臨終者接受照護 |
| 17. I know how to navigate funeral services and options | 17. I know how to navigate funeral services and options | 17. 我知道如何尋求和選擇殯儀／喪葬服務 |
| 18. I know how to access palliative care in my area | 18. I know how to access palliative care in my area | 18. 我知道如何在我所在的地區獲得紓緩／安寧療護服務 |
| 19. I have sufficient understanding of illness trajectories to make informed decisions around medical treatments available and how that will shape quality of end of life | 19. When I am seriously ill, I have sufficient understanding of illness trajectories to make informed decisions around medical treatments available and how that will shape quality of end of life | 19. 如果我患有重病，當我對疾病過程有足夠的了解，便可在知曉治療方案的情況下作出決定，以及知道該決定將如何影響末期生命品質／質量 |
| 20. I know about the contribution the cemetery staff can make at end of life | 20. I know what the funeral/ cemetery staff can help at end of life | 20. 我知道殯儀／喪葬工作人員在人們臨終時可以起到的幫助 |
| **Community Knowledge** (2 Subscales) | **Community Knowledge** | **社區知識（2個分量表）** |
| **Others can help me provide end of life care** | **Others can help me provide end of life care** | **其他能為我提供生命末期照護支持的人** |
| Please rate your level of agreement with the following statements. *If I were to provide end of life care for someone, I know people who could help me* (on a scale of 1-5 between *Strongly disagree* to *Strongly agree*) | Please rate your level of agreement with the following statements. *If I were to provide end of life care for someone, I know people who could help me* (on a scale of 1-5 between *Strongly disagree* to *Strongly agree*) | 請評價你對以下每項陳述的同意程度。*假如我要為某人提供生命末期照護，我知道誰可以幫助我*（在1-5的範圍內，介乎於*非常不同意*至*非常同意*之間） |
| 21. Access community support | 21. Access community support | 21. 獲得社區支持 |
| 22. Provide day to day care for the dying person | 22. Provide day to day care for the dying person | 22. 為臨終者提供日常照顧 |
| 23. Access equipment required for care | 23. Access equipment required for care | 23. 獲得所需要的照護設備 |
| 24. Access culturally appropriate support | 24. Access appropriate support in line with Chinese culture | 24. 獲得符合華人文化的支持 |
| 25. Access emotional support for myself | 25. Access emotional support for myself | 25. 為自己獲得情感支持 |
| **Support groups in my community** (2) | **Community support group** | **社區中的支持小組（2）** |
| Please rate your level of agreement with the following statements. *There are support groups in my community for* (on a scale of 1-5 between *Strongly disagree* to *Strongly agree*) | Please rate your level of agreement with the following statements. *There are support groups in my community for* (on a scale of 1-5 between *Strongly disagree* to *Strongly agree*) | 請評價您對以下陳述的同意程度。*在我的社區中有一些支持小組為下列人群提供支持*（在1-5 的範圍內（在1-5的範圍內，介乎於*非常不同意*至*非常同意*之間） |
| 26. People with life threatening illnesses | 26. People with life threatening illnesses | 26. 患有危重疾病的人 |
| 27. People who are dying | 27. People who are dying | 27. 臨終者 |
| 28. Carers for people who are dying | 28. Carers for people who are dying | 28. 臨終者的照顧者 |
| 29. People who are grieving | 29. People who are grieving | 29. 哀傷者 |

*Highlights in red are the difference between the original and the translated version.
