# Supplementary material for: The Death Literacy Index: translation, cultural adaptation, and validation of the Chinese version
Source: Front Public Health. 2023 May 11;11:1140475. doi: 10.3389/fpubh.2023.1140475 (PMC10213892; doi:10.3389/fpubh.2023.1140475)
Supplement: Supplementary file 2 [file Data_Sheet_2.DOCX]

**Supplement 2**

**Exploratory factor analysis and convergent validity of the five-factor translated DLI (*n*=3221)***

|  | Factor Loading | | | | | Communalities |
| --- | --- | --- | --- | --- | --- | --- |
|  | F1 | F2 | F3 | F4 | F5 |  |
| 17. I know how to navigate funeral services and options | 0.80 |  |  |  |  | 0.71 |
| 15. I feel confident in knowing what documents you need to complete in preparing for death | 0.78 |  |  |  |  | 0.68 |
| 16. I know how to navigate the health care system to support a dying person to receive care | 0.76 |  |  |  |  | 0.69 |
| 18. I know how to access palliative care in my area | 0.73 |  |  |  |  | 0.67 |
| 14. I know the law regarding dying at home | 0.72 |  |  |  |  | 0.61 |
| 20. I know what the funeral/ cemetery staff can help at end of life | 0.62 |  |  |  |  | 0.54 |
| 19. When I am seriously ill, I have sufficient understanding of illness trajectories to make informed decisions around medical treatments available and how that will shape quality of end of life | 0.59 |  |  |  |  | 0.49 |
| 28. Carers for people who are dying |  | 0.91 |  |  |  | 0.90 |
| 27. People who are dying |  | 0.91 |  |  |  | 0.89 |
| 26. People with life threatening illnesses |  | 0.89 |  |  |  | 0.86 |
| 29. People who are grieving |  | 0.88 |  |  |  | 0.85 |
| 22. Provide day to day care for the dying person | 0.57 | 0.64 |  |  |  | 0.75 |
| 23. Access equipment required for care | 0.59 | 0.64 |  |  |  | 0.77 |
| 24. Access support in line with Chinese culture | 0.58 | 0.63 |  |  |  | 0.74 |
| 25. Access emotional support for myself | 0.59 | 0.63 |  |  |  | 0.66 |
| 21. Access to community support | 0.52 | 0.58 |  |  |  | 0.76 |
| 11. Increased my life wisdom and understanding |  |  | 0.85 |  |  | 0.81 |
| 12. Made me more compassionate toward myself |  |  | 0.84 |  |  | 0.79 |
| 13. Provided me with skills and strategies when facing similar challenges in the future |  |  | 0.82 |  |  | 0.79 |
| 10. Led me to re-evaluate what is important and not important in life |  |  | 0.77 |  |  | 0.70 |
| 9. Increased my emotional strength to help others with death and dying processes |  |  | 0.72 |  |  | 0.67 |
| 7. Lifting a person or assisting to transfer them |  |  |  | 0.85 |  | 0.80 |
| 5. Feeding a person or assisting them to eat |  |  |  | 0.83 |  | 0.80 |
| 6. Bathing a person |  |  |  | 0.81 |  | 0.71 |
| 8. Administering injections |  |  |  | 0.72 |  | 0.58 |
| 2. Talk about death, dying or grieving to a child |  |  |  |  | 0.82 | 0.73 |
| 1. Talk about death, dying or grieving to a close friend |  |  |  |  | 0.78 | 0.71 |
| 4. Talk to a GP about support at home or in their place of care for a dying person |  |  |  |  | 0.70 | 0.62 |
| 3. Talk to a newly bereaved person about their loss |  |  |  |  | 0.61 | 0.47 |
| Eigenvalues | 5.78 | 5.70 | 3.79 | 3.03 | 2.45 |  |
| % of Variance | 19.93 | 19.67 | 13.07 | 10.46 | 8.46 |  |
| % of Cumulative Variance | 19.93 | 39.60 | 52.67 | 63.13 | 71.59 |  |
| Cronbach's alpha | 0.91 | 0.96 | 0.91 | 0.85 | 0.76 | 0.94 |

* Only loadings ≥ 0.40 in the items are shown in the table.

F1: Factual Knowledge; F2: Community knowledge; F3: Experiential knowledge; F4: Hands on care F5: Talking support.
